# [1-(4-Chloro­phen­yl)-5-hy­droxy-3-phenyl-1*H*-pyrazol-4-yl](thio­phen-2-yl)methanone

**DOI:** 10.1107/S1600536812019253

**Published:** 2012-05-05

**Authors:** Gui-Ming Deng, He-Ming Zhang, Lin-Qi Ou-Yang, Qiao-Zhen Tong, Shan Li

**Affiliations:** a MOE Key Laboratory of Laser Life Science & Institute of Laser Life Science, College of Biophotonics, South China Normal University, Guangzhou 510631, People’s Republic of China; b The First Affiliated Hospital of Hunan University of Chinese Medicine, Changsha 410007, People’s Republic of China

## Abstract

In the title compound, C_20_H_13_ClN_2_O_2_S, the chloro­phenyl, phenyl and thienoyl rings are oriented at dihedral angles 17.84 (7), 53.13 (8) and 34.03 (8)°, respectively, to the central pyrazole ring. An intra­molecular O—H⋯O hydrogen bond occurs. In the crystal, pairs of bifurcated O—H⋯O hydrogen bonds link mol­ecules into inversion dimers with *R*_2_^2^(12) graph-set motifs.

## Related literature

For general background to pyrazolone and its complexes, see: Li *et al.* (2000 ▶); Kimata *et al.* (2007 ▶). For related structures, see: Li *et al.* (2007 ▶); Cingolani *et al.* (2004 ▶); Holzer *et al.* (1999 ▶). For the synthesis of the title compound, see: Jensen (1959 ▶). For bond-length data, see: Allen *et al.* (1987 ▶); Foces-Foces *et al.* (1997 ▶). For graph-set motifs, see: Etter *et al.* (1990 ▶).
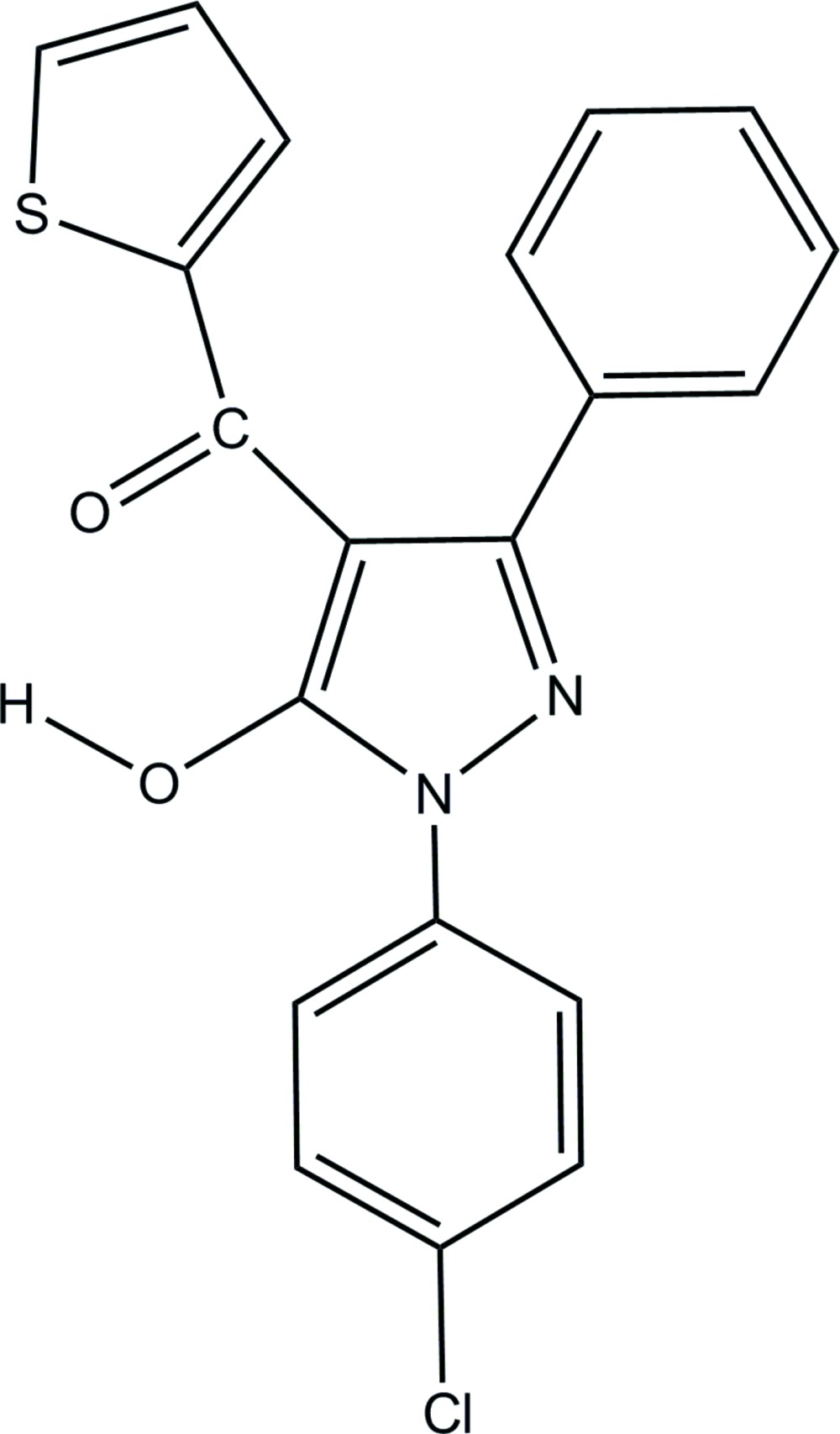


## Experimental

### 

#### Crystal data

C_20_H_13_ClN_2_O_2_S
*M* *_r_* = 380.84
Monoclinic, 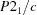

*a* = 6.0686 (2) Å
*b* = 18.6887 (5) Å
*c* = 14.9734 (4) Å
β = 91.559 (1)°
*V* = 1697.57 (9) Å^3^
*Z* = 4
Mo *K*α radiation
μ = 0.37 mm^−1^
*T* = 296 K
0.22 × 0.20 × 0.18 mm

#### Data collection

Bruker SMART CCD diffractometer
20900 measured reflections
3351 independent reflections
3072 reflections with *I* > 2σ(*I*)
*R*_int_ = 0.024

#### Refinement

*R*[*F* ^2^ > 2σ(*F* ^2^)] = 0.031
*wR*(*F* ^2^) = 0.088
*S* = 1.06
3351 reflections
235 parameters
H-atom parameters constrained
Δρ_max_ = 0.31 e Å^−3^
Δρ_min_ = −0.31 e Å^−3^

### 

Data collection: *SMART* (Bruker, 1998 ▶); cell refinement: *SAINT* (Bruker, 1998 ▶); data reduction: *SAINT*; program(s) used to solve structure: *SHELXTL* (Sheldrick, 2008 ▶); program(s) used to refine structure: *SHELXTL* ; molecular graphics: *SHELXTL*; software used to prepare material for publication: *SHELXTL*.

## Supplementary Material

Crystal structure: contains datablock(s) I, global. DOI: 10.1107/S1600536812019253/xu5526sup1.cif

Structure factors: contains datablock(s) I. DOI: 10.1107/S1600536812019253/xu5526Isup2.hkl

Supplementary material file. DOI: 10.1107/S1600536812019253/xu5526Isup3.cml

Additional supplementary materials:  crystallographic information; 3D view; checkCIF report

## Figures and Tables

**Table 1:** Hydrogen-bond geometry (Å, °)

*D*—H⋯*A*	*D*—H	H⋯*A*	*D*⋯*A*	*D*—H⋯*A*
O1—H1⋯O2	0.82	2.08	2.7233 (15)	135
O1—H1⋯O2^i^	0.82	2.12	2.7964 (15)	140

Symmetry code: (i) 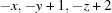.

**Fig. 1.**
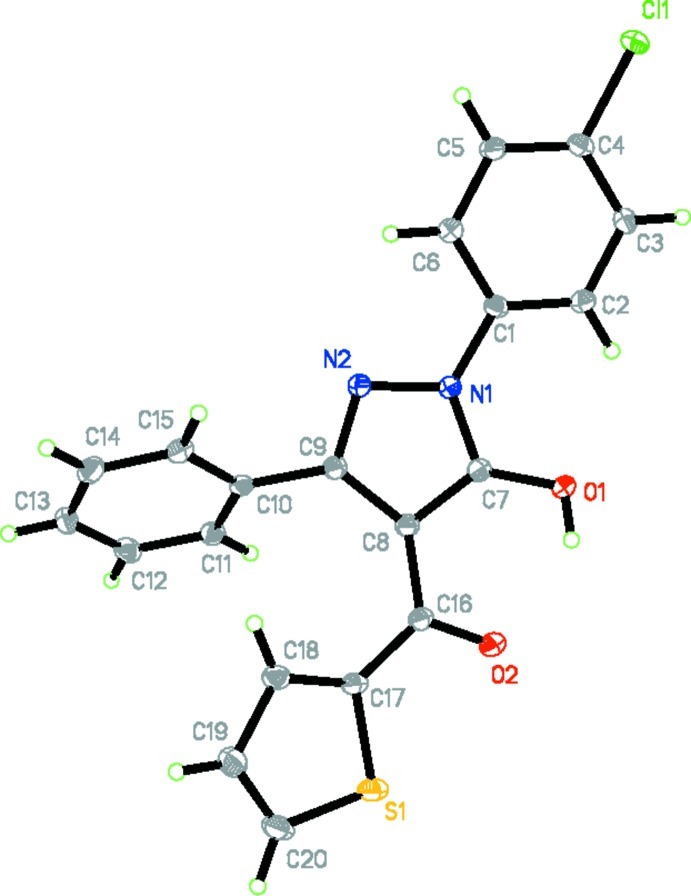
The molecular structure of (I) (thermal ellipsoids are shown at 30% probability levels).

**Fig. 2.**
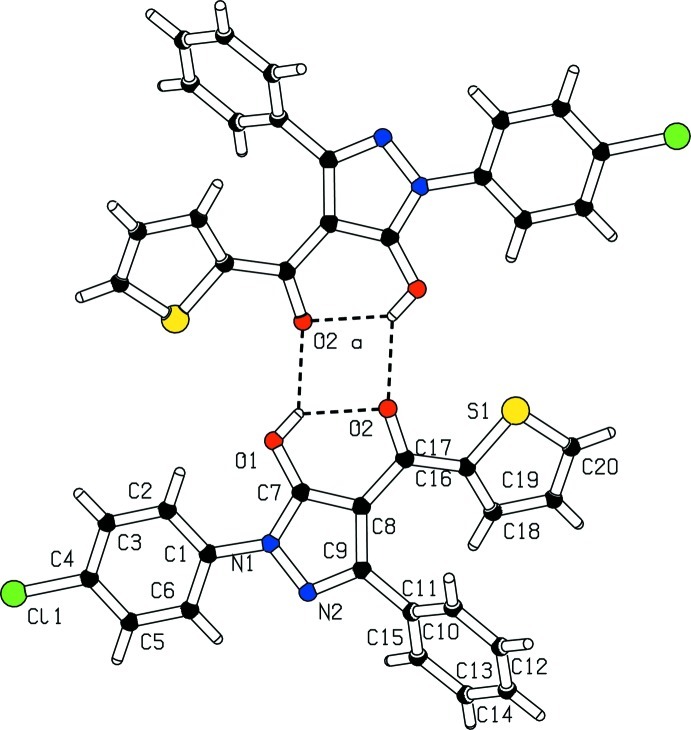
The structure of a dimer of (I).

## supplementary crystallographic information

### Comment

Pyrazolone, as a prominent structural motif, is found in numerous active compounds. Due to the easy preparation and its rich biological activity of broad-spectrum antibacterial action, antitumor, antisepsis(Kimata *et al.*, 2007). Pyrazolone and its complexes have both received considerable attention in coordination chemistry and medicinal chemistry(Li *et al.*, 2000).We report here the crystal structure of a new 4-heterocyclic acylpyrazolone (Fig. 1).

The chlorophenyl ring is slightly twisted by 17.84 (1) with respect to the pyrazolone ring, whereas the benzene and 2-thienoyl rings make dihedral angles of 53.13 (3) and 34.03 (1), respectively, with the pyrazolone (Fig. 1). The clear evidence of the hydroxyl H atom in the difference Fourier synthesis and the absence of any residual electron density in the vicinity of C7 confirm that compound (I) crystallizes as a pure enol tautomer and that no desmotropism is present (Foces-Foces *et al.*, 1997).

The molecular structure of (I) is shown in Fig. 1, and the intermolecular O—H···O hydrogen bond (Table 1) results in the formation of a dimer with an *R*_2_^2^(12) graph-set motif(Etter *et al.*, 1990)(Fig. 2.). The bond lengths and angles are within normal ranges (Allen *et al.*, 1987). Similar crystal structure of some compounds have been reported (Li *et al.*, 2007; Cingolani *et al.*, 2004; Holzer *et al.*., 1999).

### Experimental

Compound (I) was synthesized and purified according to the method proposed by Jensen (1959). (yield 72.4%.). Analysis, required for C_20_H_13_ClN_2_O_2_S: C 63.07, H 3.44, N 9.31%, S 8.42; found: C 63.01, H 3.53, N 9.34%, S 8.47. Block-like yellow single crystals of (I) were grown from an ethanol solution by slow evaporation for several weeks.

### Refinement

The hydroxyl H atom was located in a difference Fourier map and refined as riding, with O—H distance restraint of 0.82 (1) Å and with *U*_iso_(H) = 1.5U_eq_(O). Other H atoms were placed in calculated positions and constrained to ride on their parent atoms, with C—H = 0.93 Å, and with *U*_iso_(H) = 1.2U_eq_(C).

### Crystal data

**Table:**

C_20_H_13_ClN_2_O_2_S	*F*(000) = 784.0
*M**_r_* = 380.84	*D*_x_ = 1.490 Mg m^−^^3^
Monoclinic, *P*2_1_/*c*	Mo *K*α radiation, λ = 0.71073 Å
Hall symbol: -P 2ybc	Cell parameters from 9988 reflections
*a* = 6.0686 (2) Å	θ = 3.1–28.2°
*b* = 18.6887 (5) Å	µ = 0.37 mm^−^^1^
*c* = 14.9734 (4) Å	*T* = 296 K
β = 91.559 (1)°	Block, yellow
*V* = 1697.57 (9) Å^3^	0.22 × 0.20 × 0.18 mm
*Z* = 4	

### Data collection

**Table:**

Bruker SMART CCD diffractometer	3072 reflections with *I* > 2σ(*I*)
Radiation source: fine-focus sealed tube	*R*_int_ = 0.024
Graphite monochromator	θ_max_ = 26.0°, θ_min_ = 1.7°
ω scans	*h* = −7→7
20900 measured reflections	*k* = −23→22
3351 independent reflections	*l* = −18→18

### Refinement

**Table:**

Refinement on *F*^2^	Primary atom site location: structure-invariant direct methods
Least-squares matrix: full	Secondary atom site location: difference Fourier map
*R*[*F*^2^ > 2σ(*F*^2^)] = 0.031	Hydrogen site location: inferred from neighbouring sites
*wR*(*F*^2^) = 0.088	H-atom parameters constrained
*S* = 1.06	*w* = 1/[σ^2^(*F*_o_^2^) + (0.0494*P*)^2^ + 0.8*P*] where *P* = (*F*_o_^2^ + 2*F*_c_^2^)/3
3351 reflections	(Δ/σ)_max_ = 0.002
235 parameters	Δρ_max_ = 0.31 e Å^−^^3^
0 restraints	Δρ_min_ = −0.31 e Å^−^^3^

### Special details

**Table:**

Geometry. All e.s.d.'s (except the e.s.d. in the dihedral angle between two l.s. planes) are estimated using the full covariance matrix. The cell e.s.d.'s are taken into account individually in the estimation of e.s.d.'s in distances, angles and torsion angles; correlations between e.s.d.'s in cell parameters are only used when they are defined by crystal symmetry. An approximate (isotropic) treatment of cell e.s.d.'s is used for estimating e.s.d.'s involving l.s. planes.
Refinement. Refinement of *F*^2^ against ALL reflections. The weighted *R*-factor *wR* and goodness of fit *S* are based on *F*^2^, conventional *R*-factors *R* are based on *F*, with *F* set to zero for negative *F*^2^. The threshold expression of *F*^2^ > σ(*F*^2^) is used only for calculating *R*-factors(gt) *etc*. and is not relevant to the choice of reflections for refinement. *R*-factors based on *F*^2^ are statistically about twice as large as those based on *F*, and *R*- factors based on ALL data will be even larger.

### Fractional atomic coordinates and isotropic or equivalent isotropic displacement parameters (Å^2^)

**Table:**

	*x*	*y*	*z*	*U*_iso_*/*U*_eq_	
C1	0.7121 (2)	0.56022 (8)	0.81785 (9)	0.0221 (3)	
C2	0.6399 (2)	0.49761 (8)	0.77634 (10)	0.0261 (3)	
H2	0.5030	0.4784	0.7892	0.031*	
C3	0.7742 (3)	0.46391 (8)	0.71551 (10)	0.0269 (3)	
H3	0.7280	0.4219	0.6876	0.032*	
C4	0.9765 (2)	0.49323 (8)	0.69683 (10)	0.0241 (3)	
C5	1.0483 (2)	0.55577 (8)	0.73728 (10)	0.0258 (3)	
H5	1.1843	0.5752	0.7235	0.031*	
C6	0.9166 (2)	0.58925 (8)	0.79828 (10)	0.0255 (3)	
H6	0.9643	0.6311	0.8263	0.031*	
C7	0.4045 (2)	0.57557 (8)	0.92592 (9)	0.0224 (3)	
C8	0.3346 (2)	0.63263 (8)	0.97842 (9)	0.0222 (3)	
C9	0.4825 (2)	0.68917 (8)	0.95717 (9)	0.0223 (3)	
C10	0.4907 (2)	0.76450 (8)	0.98822 (9)	0.0218 (3)	
C11	0.3064 (2)	0.80840 (8)	0.98391 (10)	0.0252 (3)	
H11	0.1744	0.7912	0.9594	0.030*	
C12	0.3185 (3)	0.87803 (8)	1.01608 (11)	0.0301 (3)	
H12	0.1949	0.9074	1.0125	0.036*	
C13	0.5140 (3)	0.90380 (8)	1.05335 (11)	0.0311 (3)	
H13	0.5209	0.9501	1.0760	0.037*	
C14	0.6989 (3)	0.86062 (9)	1.05681 (10)	0.0301 (3)	
H14	0.8305	0.8779	1.0817	0.036*	
C15	0.6887 (2)	0.79162 (8)	1.02331 (10)	0.0257 (3)	
H15	0.8146	0.7632	1.0242	0.031*	
C16	0.1539 (2)	0.62147 (8)	1.03925 (10)	0.0236 (3)	
C17	0.1226 (2)	0.66529 (8)	1.11888 (10)	0.0234 (3)	
C18	0.2674 (3)	0.71042 (8)	1.16592 (10)	0.0281 (3)	
H18	0.4099	0.7209	1.1487	0.034*	
C19	0.1698 (3)	0.73824 (9)	1.24312 (11)	0.0363 (4)	
H19	0.2418	0.7694	1.2826	0.044*	
C20	−0.0405 (3)	0.71486 (9)	1.25382 (11)	0.0367 (4)	
H20	−0.1283	0.7290	1.3006	0.044*	
Cl1	1.14533 (6)	0.45042 (2)	0.62109 (2)	0.03082 (12)	
N1	0.5794 (2)	0.59767 (6)	0.87952 (8)	0.0233 (3)	
N2	0.6279 (2)	0.66882 (6)	0.89840 (8)	0.0246 (3)	
O1	0.33334 (17)	0.50886 (5)	0.91919 (7)	0.0258 (2)	
H1	0.2308	0.5029	0.9530	0.039*	
O2	0.02678 (19)	0.57078 (6)	1.02388 (8)	0.0355 (3)	
S1	−0.12432 (6)	0.65709 (2)	1.17238 (3)	0.03080 (12)	

### Atomic displacement parameters (Å^2^)

**Table:**

	*U*^11^	*U*^22^	*U*^33^	*U*^12^	*U*^13^	*U*^23^
C1	0.0219 (7)	0.0224 (7)	0.0221 (7)	0.0033 (5)	0.0034 (5)	0.0018 (5)
C2	0.0230 (7)	0.0248 (7)	0.0308 (7)	−0.0007 (6)	0.0056 (6)	0.0006 (6)
C3	0.0303 (8)	0.0222 (7)	0.0283 (7)	0.0008 (6)	0.0039 (6)	−0.0027 (6)
C4	0.0258 (7)	0.0246 (7)	0.0221 (7)	0.0063 (6)	0.0047 (5)	0.0018 (5)
C5	0.0215 (7)	0.0282 (8)	0.0278 (7)	−0.0003 (6)	0.0049 (6)	0.0009 (6)
C6	0.0250 (7)	0.0242 (7)	0.0275 (7)	−0.0010 (6)	0.0029 (6)	−0.0013 (6)
C7	0.0215 (7)	0.0225 (7)	0.0233 (7)	−0.0011 (5)	0.0020 (5)	0.0015 (5)
C8	0.0214 (7)	0.0228 (7)	0.0226 (7)	0.0006 (5)	0.0027 (5)	0.0007 (5)
C9	0.0215 (7)	0.0209 (7)	0.0245 (7)	0.0012 (5)	0.0022 (5)	0.0021 (5)
C10	0.0241 (7)	0.0210 (7)	0.0206 (6)	−0.0004 (5)	0.0056 (5)	0.0024 (5)
C11	0.0222 (7)	0.0281 (8)	0.0254 (7)	0.0006 (6)	0.0009 (6)	−0.0005 (6)
C12	0.0309 (8)	0.0263 (8)	0.0335 (8)	0.0065 (6)	0.0054 (6)	−0.0001 (6)
C13	0.0410 (9)	0.0233 (7)	0.0293 (8)	−0.0034 (7)	0.0074 (7)	−0.0042 (6)
C14	0.0288 (8)	0.0323 (8)	0.0291 (8)	−0.0094 (6)	0.0004 (6)	0.0010 (6)
C15	0.0218 (7)	0.0263 (7)	0.0290 (7)	0.0005 (6)	0.0028 (6)	0.0054 (6)
C16	0.0221 (7)	0.0232 (7)	0.0257 (7)	0.0011 (6)	0.0038 (6)	0.0031 (6)
C17	0.0222 (7)	0.0240 (7)	0.0242 (7)	0.0038 (5)	0.0052 (6)	0.0049 (6)
C18	0.0340 (8)	0.0273 (7)	0.0231 (7)	0.0026 (6)	0.0070 (6)	0.0042 (6)
C19	0.0485 (10)	0.0318 (8)	0.0288 (8)	−0.0009 (7)	0.0050 (7)	−0.0022 (7)
C20	0.0466 (10)	0.0346 (9)	0.0297 (8)	0.0092 (7)	0.0148 (7)	0.0006 (7)
Cl1	0.0322 (2)	0.0302 (2)	0.0306 (2)	0.00225 (15)	0.01222 (15)	−0.00420 (14)
N1	0.0238 (6)	0.0194 (6)	0.0269 (6)	−0.0005 (5)	0.0062 (5)	−0.0010 (5)
N2	0.0248 (6)	0.0197 (6)	0.0298 (6)	−0.0007 (5)	0.0069 (5)	−0.0011 (5)
O1	0.0246 (5)	0.0227 (5)	0.0305 (5)	−0.0047 (4)	0.0081 (4)	−0.0014 (4)
O2	0.0344 (6)	0.0343 (6)	0.0386 (6)	−0.0122 (5)	0.0144 (5)	−0.0069 (5)
S1	0.0272 (2)	0.0344 (2)	0.0313 (2)	0.00437 (15)	0.01022 (16)	0.00459 (15)

### Geometric parameters (Å, º)

**Table:**

C1—C2	1.390 (2)	C11—C12	1.389 (2)
C1—C6	1.393 (2)	C11—H11	0.9300
C1—N1	1.4254 (18)	C12—C13	1.384 (2)
C2—C3	1.389 (2)	C12—H12	0.9300
C2—H2	0.9300	C13—C14	1.382 (2)
C3—C4	1.380 (2)	C13—H13	0.9300
C3—H3	0.9300	C14—C15	1.384 (2)
C4—C5	1.381 (2)	C14—H14	0.9300
C4—Cl1	1.7432 (14)	C15—H15	0.9300
C5—C6	1.380 (2)	C16—O2	1.2393 (19)
C5—H5	0.9300	C16—C17	1.463 (2)
C6—H6	0.9300	C17—C18	1.395 (2)
C7—O1	1.3224 (17)	C17—S1	1.7253 (14)
C7—N1	1.3491 (18)	C18—C19	1.412 (2)
C7—C8	1.397 (2)	C18—H18	0.9300
C8—C9	1.428 (2)	C19—C20	1.363 (3)
C8—C16	1.4595 (19)	C19—H19	0.9300
C9—N2	1.3191 (19)	C20—S1	1.6965 (19)
C9—C10	1.483 (2)	C20—H20	0.9300
C10—C11	1.387 (2)	N1—N2	1.3892 (17)
C10—C15	1.394 (2)	O1—H1	0.8200
			
C2—C1—C6	120.36 (13)	C13—C12—C11	120.18 (14)
C2—C1—N1	121.76 (13)	C13—C12—H12	119.9
C6—C1—N1	117.86 (13)	C11—C12—H12	119.9
C3—C2—C1	119.44 (14)	C14—C13—C12	119.87 (15)
C3—C2—H2	120.3	C14—C13—H13	120.1
C1—C2—H2	120.3	C12—C13—H13	120.1
C4—C3—C2	119.59 (14)	C13—C14—C15	120.14 (14)
C4—C3—H3	120.2	C13—C14—H14	119.9
C2—C3—H3	120.2	C15—C14—H14	119.9
C3—C4—C5	121.24 (14)	C14—C15—C10	120.32 (14)
C3—C4—Cl1	119.44 (12)	C14—C15—H15	119.8
C5—C4—Cl1	119.33 (11)	C10—C15—H15	119.8
C6—C5—C4	119.52 (14)	O2—C16—C8	117.89 (13)
C6—C5—H5	120.2	O2—C16—C17	119.04 (13)
C4—C5—H5	120.2	C8—C16—C17	123.02 (13)
C5—C6—C1	119.86 (14)	C18—C17—C16	131.02 (13)
C5—C6—H6	120.1	C18—C17—S1	111.24 (11)
C1—C6—H6	120.1	C16—C17—S1	117.53 (11)
O1—C7—N1	120.61 (13)	C17—C18—C19	111.34 (15)
O1—C7—C8	131.21 (13)	C17—C18—H18	124.3
N1—C7—C8	108.13 (12)	C19—C18—H18	124.3
C7—C8—C9	103.72 (12)	C20—C19—C18	113.14 (16)
C7—C8—C16	119.13 (13)	C20—C19—H19	123.4
C9—C8—C16	137.08 (13)	C18—C19—H19	123.4
N2—C9—C8	111.79 (13)	C19—C20—S1	112.52 (13)
N2—C9—C10	117.74 (13)	C19—C20—H20	123.7
C8—C9—C10	130.44 (13)	S1—C20—H20	123.7
C11—C10—C15	119.22 (14)	C7—N1—N2	110.71 (11)
C11—C10—C9	121.78 (13)	C7—N1—C1	130.51 (12)
C15—C10—C9	119.00 (13)	N2—N1—C1	118.78 (11)
C10—C11—C12	120.21 (14)	C9—N2—N1	105.65 (11)
C10—C11—H11	119.9	C7—O1—H1	109.5
C12—C11—H11	119.9	C20—S1—C17	91.72 (8)
			
C6—C1—C2—C3	0.4 (2)	C9—C10—C15—C14	−176.86 (13)
N1—C1—C2—C3	178.83 (13)	C7—C8—C16—O2	−21.5 (2)
C1—C2—C3—C4	−0.3 (2)	C9—C8—C16—O2	162.36 (17)
C2—C3—C4—C5	−0.2 (2)	C7—C8—C16—C17	155.84 (14)
C2—C3—C4—Cl1	179.53 (12)	C9—C8—C16—C17	−20.3 (3)
C3—C4—C5—C6	0.7 (2)	O2—C16—C17—C18	159.46 (16)
Cl1—C4—C5—C6	−179.03 (12)	C8—C16—C17—C18	−17.8 (2)
C4—C5—C6—C1	−0.6 (2)	O2—C16—C17—S1	−14.63 (19)
C2—C1—C6—C5	0.1 (2)	C8—C16—C17—S1	168.10 (11)
N1—C1—C6—C5	−178.40 (13)	C16—C17—C18—C19	−175.79 (15)
O1—C7—C8—C9	178.08 (15)	S1—C17—C18—C19	−1.42 (17)
N1—C7—C8—C9	0.69 (16)	C17—C18—C19—C20	0.0 (2)
O1—C7—C8—C16	0.8 (2)	C18—C19—C20—S1	1.4 (2)
N1—C7—C8—C16	−176.63 (12)	O1—C7—N1—N2	−178.57 (12)
C7—C8—C9—N2	−0.31 (17)	C8—C7—N1—N2	−0.85 (16)
C16—C8—C9—N2	176.25 (16)	O1—C7—N1—C1	1.4 (2)
C7—C8—C9—C10	177.61 (14)	C8—C7—N1—C1	179.14 (14)
C16—C8—C9—C10	−5.8 (3)	C2—C1—N1—C7	18.8 (2)
N2—C9—C10—C11	126.50 (15)	C6—C1—N1—C7	−162.77 (14)
C8—C9—C10—C11	−51.3 (2)	C2—C1—N1—N2	−161.23 (13)
N2—C9—C10—C15	−54.02 (19)	C6—C1—N1—N2	17.22 (19)
C8—C9—C10—C15	128.16 (17)	C8—C9—N2—N1	−0.18 (16)
C15—C10—C11—C12	−1.4 (2)	C10—C9—N2—N1	−178.39 (12)
C9—C10—C11—C12	178.06 (13)	C7—N1—N2—C9	0.64 (16)
C10—C11—C12—C13	−0.6 (2)	C1—N1—N2—C9	−179.36 (12)
C11—C12—C13—C14	1.4 (2)	C19—C20—S1—C17	−1.85 (14)
C12—C13—C14—C15	−0.2 (2)	C18—C17—S1—C20	1.86 (12)
C13—C14—C15—C10	−1.8 (2)	C16—C17—S1—C20	177.08 (12)
C11—C10—C15—C14	2.6 (2)		

### Hydrogen-bond geometry (Å, º)

**Table:**

*D*—H···*A*	*D*—H	H···*A*	*D*···*A*	*D*—H···*A*
O1—H1···O2	0.82	2.08	2.7233 (15)	135
O1—H1···O2^i^	0.82	2.12	2.7964 (15)	140

Symmetry code: (i) −*x*, −*y*+1, −*z*+2.
